# Configuration of a Simple Method for Different Polyamides 6.9 Recognition by ATR-FTIR Analysis Coupled with Chemometrics

**DOI:** 10.3390/polym15153166

**Published:** 2023-07-26

**Authors:** Maria Laura Tummino, Christoforos Chrimatopoulos, Maddalena Bertolla, Cinzia Tonetti, Vasilios Sakkas

**Affiliations:** 1 Institute of Intelligent Industrial Technologies and Systems for Advanced Manufacturing, National Research Council of Italy (CNR-STIIMA), Corso G. Pella 16, 13900 Biella, Italy; 2 Department of Chemistry, School of Sciences, University of Ioannina, 45110 Ioannina, Greece; 3 Aquafil S.p.A., Via Linfano 9, 38062 Arco, Italy

**Keywords:** polyamide 6.9, chemometrics, infrared spectroscopy

## Abstract

This study proposes a simple approach for the recognition of polyamide 6.9 samples differing in impurity amounts and viscosities (modulated during the synthesis), which are parameters plausibly variable in polymers’ manufacturing processes. Infrared spectroscopy (ATR-FTIR) was combined with chemometrics, applying statistical methods to experimental data. Both non-supervised and supervised methods have been used (PCA and PLS-DA), and a predictive model that could assess the polyamide type of unknown samples was created. Chemometric tools led to a satisfying degree of discrimination among samples, and the predictive model resulted in a great classification of unknown samples with an accuracy of 88.89%. Traditional physical-chemical characterizations (such as thermal and mechanical tests) showed their limits in the univocal identification of sample types, and additionally, they resulted in time-consuming procedures and specimen destruction. The spectral modifications have been investigated to understand the main signals that are more likely to affect the discrimination process. The proposed hybrid methodology represents a potential support for quality control activities within the production sector, especially when the spectra of compounds with the same nominal composition show almost identical signals.

## 1. Introduction

Polyamides (PA), also known as nylon, are a family of widespread polymeric materials applicable in many industrial sectors. The size of the global polyamides market is estimated at USD 33.30 billion in 2020 and an expansion is expected at a compound annual growth rate of 6.2% from 2021 to 2028 [1]. Beyond the different types of polyamides available (such as 6, 66, 11, 12, etc.) that are differentiated by the distance between the amide groups, new kinds of materials are being studied and are approaching commercialization, for instance, recycled and bio-based ones [2,3,4,5]. Moreover, if the chemical composition is equal, the different processes carried out in distinct manufacturing lines or the random variations that can occur within a production chain can lead to different material characteristics [6]. This variability raises the necessity for more and more sensitive techniques of quality check recognition, possibly rapid and non-destructive. In this direction, infrared spectroscopy and, in particular, Attenuated Total Reflectance (ATR) Fourier Transform Infrared (FTIR) spectroscopy have become practical tools in the field of forensic and analytical science, as they can be used in a non-destructive way for the analysis of various samples shaped as powders, pellets, fibers, films, etc. without pre-treatment [7,8]. Much work has been published where this technique has been successfully employed along with chemometrics and multivariate statistical analyses, which give a significant and better interpretation of results [9,10,11,12].

**Figure 1:**
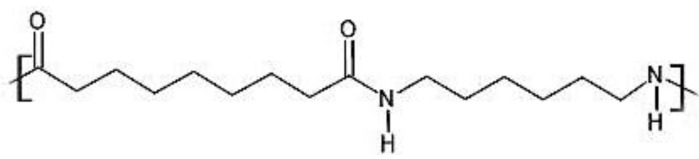
Chemical structure of polyamide 6.9.

This work is focused on polyamide 6.9 (hereinafter, PA69), which derives from hexamethylene diamine and azelaic acid (a potentially bio-based monomer) [13,14] (see Figure 1). Indeed, the development of monomers derived from bio-based sources, such as vegetable oils, has recently been boosted in the polymer production sector due to the depletion of fossil oil resources [13,15].

PA69 is characterized by ferroelectric behavior and high thermal properties close to odd polyamides and has been used in electrical applications; moreover, the presence of an odd number of carbon atoms in the constitutive unit improves the electroactive properties, and the presence of extra methylene groups enhances the water resistance, dimensional stability, and electrical properties [16,17,18,19]. Nevertheless, this material is not very frequently studied in research articles, especially as a sole component. Literature often reports on PA69 in combination with other materials. For instance, it was used in random copolymers with PA6 (to tune PA6 mechanical and thermal properties) [13,20], even blended with cellulose [21], and it was employed to create composites with hydroxyapatites as biomimetic semi-rigid implant materials destined for orthopaedic applications [19].

Therefore, to deepen the knowledge about PA69, this work can provide a more structured framework to understand its variable features and a method to easily detect them by spectroscopic and chemometric tools. The differences among the samples taken into account are related to (i) the presence of impurities originating from monomers and (ii) the relative viscosity, which is a tunable parameter in production processes (Table 1). Impurities can be connected to the synthesis of azelaic acid. For example, bio-based azelaic acid is obtained from the oxidative cleavage of vegetal-derived fatty acids (primarily oleic acid) [22], and this process can cause the presence of co-products (e.g., pelargonic acid and suberic acid) [22,23] or shorter di- and mono-carboxylic acids as by-products formed if over-oxidation or oxidative degradation occur, following a radical reaction pathway [24].

The variation of the two parameters (impurities and viscosity) implies technological consequences to be considered in a production plant. Indeed, in the literature, the presence of unreacted monomers or other contaminating agents (particularly in the case of bio-based monomers or recycled polymers) has been ascertained as a detrimental circumstance in terms of polymer properties and processing, potentially acting as chain terminators [12,25,26,27]. The modulation of viscosity is likewise crucial since it represents the measure of resistance to flow and, therefore, the more or less tendency of the melt polymer to proceed easily through the channels of the process equipment [28]. On the one hand, the viscosity has to be reduced to permit the flow (e.g., in injection molding) [28,29,30]; on the other hand, a low melt viscosity can be an issue when other manufacturing methods are required, as in the cases of film blowing or casting [31,32,33]. To the best of the authors’ knowledge, this is the first time that chemometrics has been applied to PA69 to discriminate samples with such different characteristics. 

In general, one of the typical characterization techniques used for distinguishing polyamides among different polymers or identifying a particular polyamide type is Differential Scanning Calorimetry (DSC). Indeed, caloric effects observed in the DSC signals in specific temperature ranges, e.g., the glass transition, crystallization, and melting, allow for a certain degree of discrimination among materials [34]. However, DSC brings about the destruction of the analyzed specimen, and when comparing very similar materials, the heat-related signals can fall in a very narrow temperature range (making it difficult to distinguish them from the experimental error), and, in particular, the phenomena detectable in calorimetry are strongly influenced by the thermal history of the materials [35]. The mechanical properties of polyamides are also routinely investigated within the production chain, especially to determine the materials’ quality in relation to the particular application or process they are destined for [36]. Although the importance of these analyses is obvious, the materials generally have to be shaped to perform the tests, and such pre-treatment is time-consuming and, in the end, leads to the material being broken. Vibrational spectroscopy, like infrared spectroscopy, is another traditional method to study the functional groups of polymers and is useful for distinguishing different polymer compositions. FTIR is considered a semi-quantitative tool that produces a characteristic spectrum that corresponds to the vibrations of the bonds within molecules, acting as a chemical fingerprint of the material. The sensitivity of such a technique obviously depends on many instrumental factors and also on the mode of acquisition. For instance, transmission FTIR involves the manual creation of pellets (e.g., with KBr) that are costly, labor-intensive, and make the analyzed samples unrecoverable [37]. For this reason, ATR is a good alternative for spectral analysis. ATR is based on the phenomenon of total internal reflection and measures changes that occur in an internally reflected infrared beam that comes into contact with the sample through a zinc selenide (ZnSe) crystal or diamond. ATR analyses are carried out by placing the specimen directly on the sampling plate of the device over the optic window with the crystal, and it is then held by a compression clamp to ensure good contact between the sample and the crystal [37]. However, regardless of which kind of IR spectra acquisition is chosen, when the samples resemble each other, the discrimination of small variations in the material features requires experienced operators and time. 

Given these premises, this work aims to propose a fast and non-destructive method for different PA69 recognitions by ATR-FTIR analysis assisted by chemometric tools.

## 2. Materials and Methods

### 2.1. Synthesis and Characterisation of PA69

The starting precursors for PA69 were hexamethylene diamine (from now on HMDA), provided by Arpadis (Antwerp, Belgium), and azelaic acid (AZA), from Merck (Darmstadt, Germany). The basic procedure for PA69 polymerization and the scheme of the plant are reported in [13]. Briefly, the monomers HMDA and AZA, in a molar ratio of 1:1, were homogenized in water, forming a soluble salt, which was transferred to the autoclave, where polymerization was managed in a 4-phase cycle at different pressures that finally brought about the complete conversion of the monomers [13]. The viscosity of the mass was controlled through the power absorption of the stirrer, and when it reached the desired level, the polymer mass was pushed to the extrusion pump and forced through the spinneret, a subsequent cold-water tub, and a cutter, where the polymer pellets were obtained.

The materials prepared and selected are reported in Table 1, together with the coordinate b * of CIELAB color space as an indication of the sample aspect and the relative viscosity (RV) values. Indeed, b * represents one of the coordinates that the CIELAB color space uses to measure objective color and calculate color differences, and, in particular, negative b * corresponds with blue and positive b * corresponds with yellow [38]. The measurements were performed with a Chroma-meter CR-5 Konica Minolta (Tokyo, Japan) in reflectance mode (measure area of 8 mm diameter), in accordance with CIE No. 15 “International Commission on Illumination, Technical Report: Colorimetry, 3rd edition” and ASTM E 1164 “Standard Practice for Obtaining Spectrometric Data for Object-Color Evaluation”.

The viscosity of the different PA69 was determined by using a Ubbelohde viscometer placed in a thermostated water bath at 25 °C. The sample was dissolved in sulfuric acid (96%, Merck, Darmstadt, Germany), and the RV was calculated as the ratio between the solution and the solvent flow time. 

The purification procedure for sample P1 was carried out through the crystallization of the salt composed of the monomers HMDA and AZA, throwing away the crystallization water and adding new water for polymerization; in this way, all soluble impurities were removed.

For stress-strain measurements (tensile testing), PA69 pellets were remelted via an injection molding machine (Sandretto, Torino, Italy), in which, through a screw extruder set at a temperature around 240 °C, the molten material was injected at a high pressure of 600 bar to fill the mold and set at a temperature around 50 °C. The lab test specimens were produced in the mold cavity with a dumbbell shape (1 A type) and were then tested via a Dynamometer Instron 34 TM-10 (Instron, Torino, Italy) following ISO 527: 2019 “Plastics—Determination of tensile properties”. The same instrument was employed for the determination of the flexural modulus, following UNI EN ISO 178: 2019 “Plastics—Determination of flexural properties”. Izod and Charpy impact tests were carried out through an Instron 9050 instrument (Instron, Torino, Italy), following, respectively, ISO 180:2020 “Plastics—Determination of Izod impact strength” and ISO 179:2010 “Plastics—Determination of Charpy impact properties”.

Differential Scanning Calorimetry (DSC) analyses were conducted with a TA Instrument DSC Q20 (TA Instruments, New Castle, DE, USA) on a sample of about 10 mg under a N_2_ flow of 100 mL min^−1^. The sample was first heated from 20 to 280 °C at a rate of 20 °C min^−1^, then cooled to 0 °C and heated again to 280 °C.

### 2.2. ATR-FTIR Measures Coupled with Chemometrics

Following a previously reported methodology [11], Spectrum Two FT-IR Spectrometer adjusted with UATR Two Accessory (ATR-FTIR) (Perkin Elmer, Waltham, MA, USA) and Spectrum 10 Spectroscopy Software v. 10.5.4 (Perkin Elmer, Waltham, MA, USA) were utilized to obtain infrared spectra in the range of 4000 to 450 cm^−1^, at a resolution of 4 cm^−1^, and 32 scans at room temperature. Spectra were baseline corrected, and multiplicative scatter correction (MSC) was applied using the Spectragryph licensed application software version 1.2.15 (Dr. Friedrich Menges Software-Entwicklung, Germany).

Principal Component Analysis (PCA) and Partial Least Squares Discriminant Analysis (PLS-DA) were carried out by MATLAB (R2019a; The Mathworks, Natick, MA, USA), using the total spectral range of 4000 to 450 cm^−1^. Pre-processed ATR-FTIR spectra were imported into the software, and the calculations were run using in-house functions and routines, based on “Partial Least Squares Regression and Principal Components Regression”, MATLAB and Simulink examples on the Mathworks website [39]. To allow the validation of the classification model, ATR-FTIR data (150 spectra) were randomly subjected to data splitting into two datasets (70:30% ratio), namely a training dataset (105 spectra) and a test dataset (45 spectra). Both PCA and PLS-DA were performed on the training set of samples to create a suitable predictive model that could assess the polyamide type of the “unknown” samples. 10-fold repeated stratified cross-validation (100 repeats) was performed on the training dataset to choose the optimum number of components, minimizing the mean squared error of cross-validation (MSECV) and avoiding an overfitting phenomenon.

## 3. Results and Discussion

Starting from the observation of the pellet appearance, the purified sample (P1) was neatly less yellow than its corresponding non-purified specimen (NP), and the sample with the highest viscosity (V3) showed a less prominent coloration. These empirical considerations were supported by the estimation of the color through the CIELAB space coordinates (Table 1). From the comparison between NP and P1, the presence of AZA-derived by-products, although at low concentrations, is the reason for such a phenomenon, as already pointed out by Vassoi et al. [23], and it was confirmed by the intensely yellow coloration of the aqueous medium used in the purification procedure. In the case of V3 with respect to V1 and V2, the yellow shade fading is imputable to the larger ratio of polymer concentration out of impurities, given the proportionality between viscosity, molecular weight, and polymer concentration [40], which could have made the impurities’ coloration effect less significant.

Another screening regarded the effect of VR on the principal physical and mechanical properties. Izod and Charpy impact tests measure the energy required to break a specimen by striking a specific size bar with a pendulum and mainly differ from each other for the specimen positioning and striking point; flexural modulus is a property that is computed as the ratio of stress to strain in flexural deformation; tensile strength, strain at yield, and elongation at break are the values commonly obtained from the stress-strain measurements in tensile mode. Indeed, viscosity can be seen as an indicator of the number/entanglement of the polymeric chains, thus potentially being able to influence mechanical behavior [41,42]. It is clear from Table 2 that the samples under examination here did not demonstrate distinct differences, nor did the purified sample (data not shown), since the small amount of impurities was not able to affect the presented parameters (the associated error was below 10%). Only the Melt Volume Rate correlates to viscosity since they are obviously inversely proportional measures.

**Figure 2:**
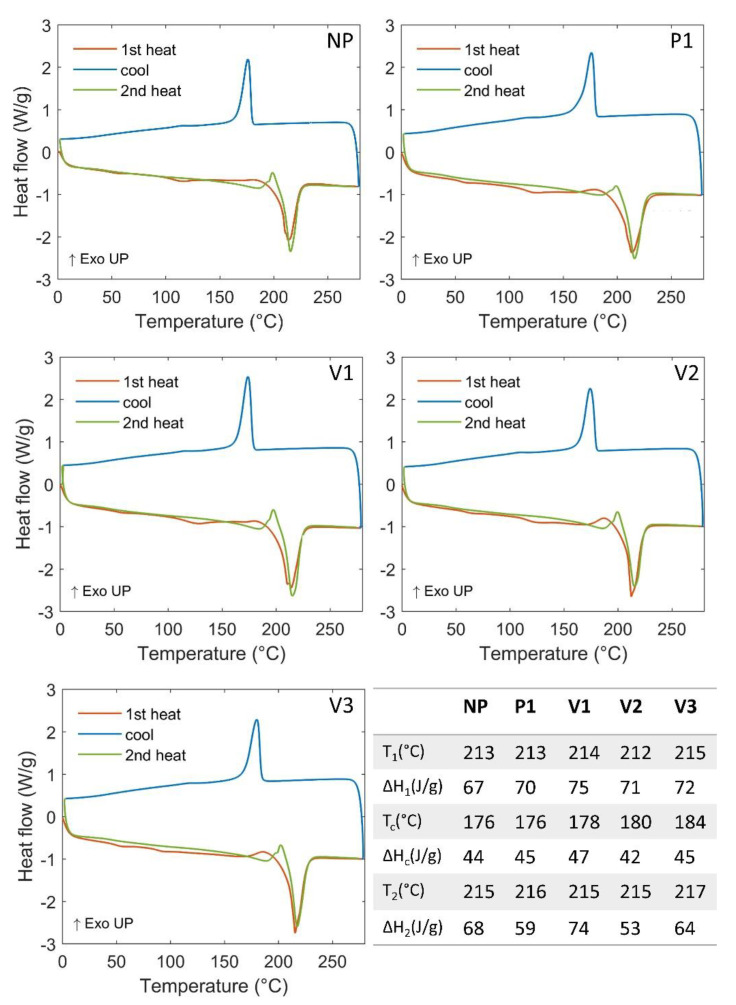
DSC analyzes outputs. In the inserted table, T_1_ and ΔH_1_ represent the temperature of the 1st heating peak and the associated enthalpy; similarly, T_c_ and ΔH_c_ and T_2_ and ΔH_2_ are related to the cooling and the 2nd heating processes, respectively.

In Figure 2, the DSC outcomes are displayed. In general, from the comparison with the literature, the thermograms obtained in this work are in good accordance with previous results [13,14]. The cycles of heating–cooling–heating are very similar for all the samples analyzed. Considering the temperatures of the peak corresponding to the main transformations and the correlated enthalpies, no remarkable differences or trends specifically attributable to the materials’ features (such as crystallinity [43]) can be highlighted.

So far, some of the methods (mechanical and thermal) traditionally employed in polymer characterization have been demonstrated to be nonfunctional in discriminating the various PA69 types univocally, and the techniques used (viscosimetry included) are all destructive.

**Figure 3:**
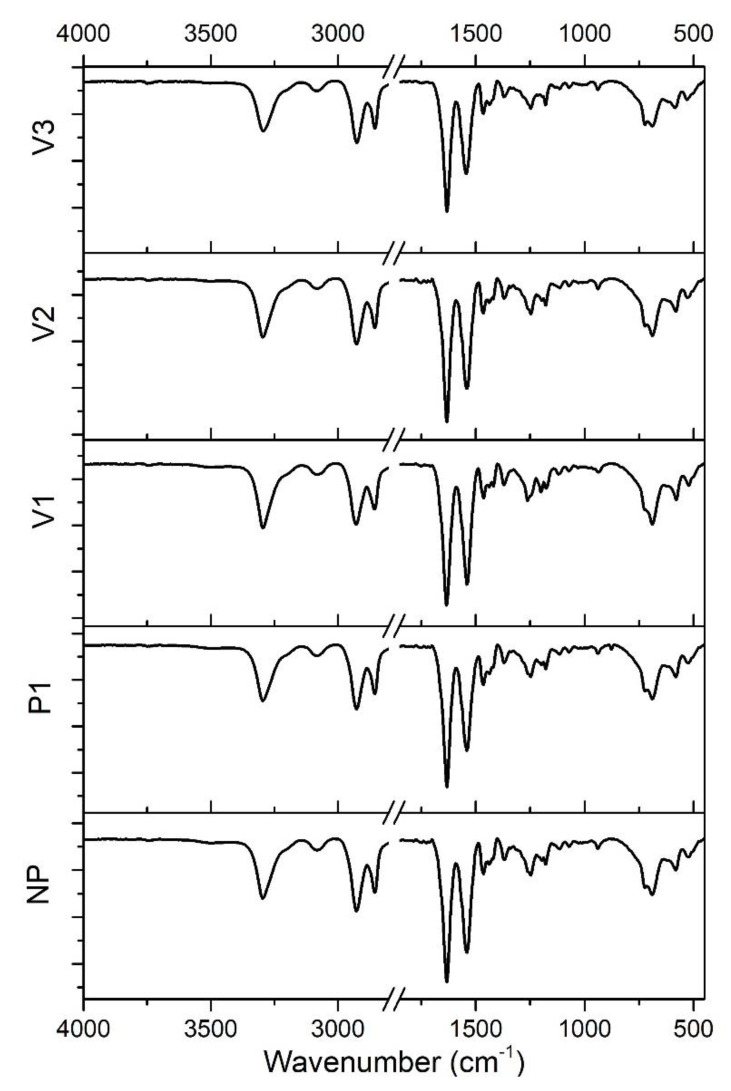
Averaged ATR-FTIR spectra of different PA69 samples (NP, P1, V1, V2, and V3) in transmittance mode.

As the core of the present study, the averaged ATR-FTIR spectra of PA69 samples from those cumulatively collected are visible in Figure 3. The spectra are almost equal to each other since they reflect the same chemical composition. More details regarding some minor peculiarities will be reported in the discussion of chemometric results. According to Tao et al. [16], the main infrared-active vibrations are related to the following:

− Around 3300 cm^−1^: stretching vibration of the amine groups (at ca. 3075 cm^−1^, the overtone of N-H bending has a signal [44]);
− At about 2930 and 2855 cm^−1^: asymmetric and symmetric stretching vibration peaks of methylene (-CH_2_-), respectively;
− Around 1634 cm^−1^ and 1542 cm^−1^: amide I [ν(C=O)] and amide II [δ(N-H), ν(C-N)] bands, respectively;
− Peaks appearing at 1465 cm^−1^ and 1370 cm^−1^: C-H asymmetric bending in (-CH_2_-) or (-CH_3_-) and symmetric bending in (-CH_3_-), respectively;
− At about 940 cm^−1^ and 685 cm^−1^: C–C=O stretching vibration and N–H out-of-plane bending vibration, respectively;
− At ca. 580 cm^−1^ and 530 cm^−1^, similarly to other polyamides [45,46,47]: C=O out-of-plane [45,46] and skeletal bending modes, respectively [47,48].

In principle, some FTIR peaks can be distinguished on the basis of the different crystalline phases that constitute polyamides [49,50,51] and, in particular, in the case of PA69, the α and γ forms [16,52]. Indeed, the main feature of a polyamide structure is the organization of chains in well-defined, two-dimensional hydrogen-bonded sheets that are held together by van der Waals interactions in a three-dimensional lattice [53]. The chains in the α crystals are in the fully extended zigzag conformation, whereas in the γ crystals, the amide groups are rotated about 60° from that in a fully extended planar conformation, resulting in a shorter chain-axis repeat and the formation of pleated sheets (even–odd polyamides crystallize primarily in the γ form, with the transformation to α occurring by heat, strain, or solvent action). The hydrogen bonds are between antiparallel chains in α crystals and between parallel chains in γ crystals [53]. For instance, peaks related to α and γ crystalline forms, appearing respectively at about 1200 cm^−1^ and 1178 cm^−1^, are reported to be representative of the different wagging or twisting vibrations of methylene, and the absorption around 685 cm^−1^ (N–H out-of-plane bending vibration) is typical of the α form [16,46,54]. In polyamides, the peak at about 725 cm^−1^ has been attributed to the N–H out-of-plane bending vibration of γ form and CH_2_ rocking modes (with four or more C-C vibrations of bound atoms) [16,46,55,56].

**Figure 4:**
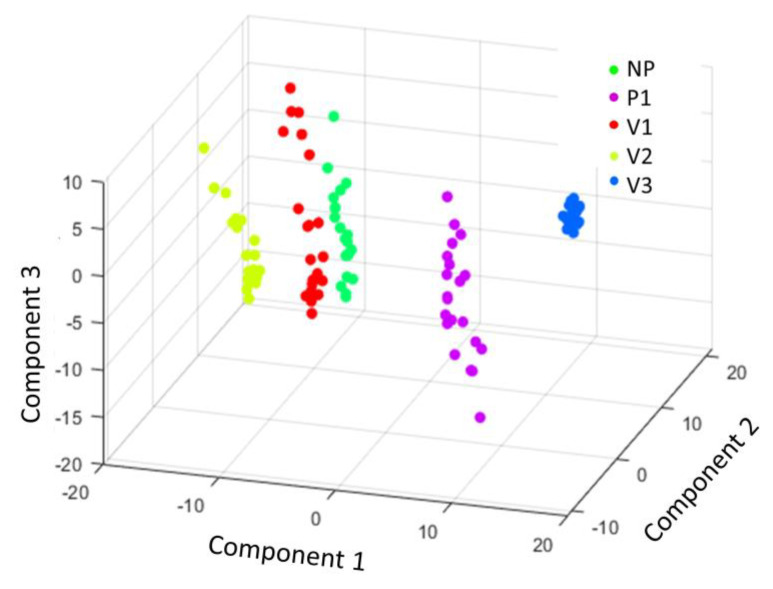
Scatter plot of principal component analysis (PCA) clustering the five groups of polyamides.

In order to examine the ability of the ATR-FTIR to differentiate the five polyamide groups, both unsupervised (PCA) and supervised (PLS-DA) chemometric models were utilized. PCA is an unsupervised method used to explain the observed variability of the samples without considering the groups to which each sample belongs. The PCA scatter score plot of the three main principal components, PCs (PC1 47.2%, PC2 24.8%, and PC3 9.9%), is presented in Figure 4, where it is displayed that the investigated materials have been well-discriminated and, especially, sample V3 showed a very small variability inside its cluster. More information about data properties and statistics is presented in the Supplementary Materials (Figures S1–S8 and Table S1).

**Figure 5:**
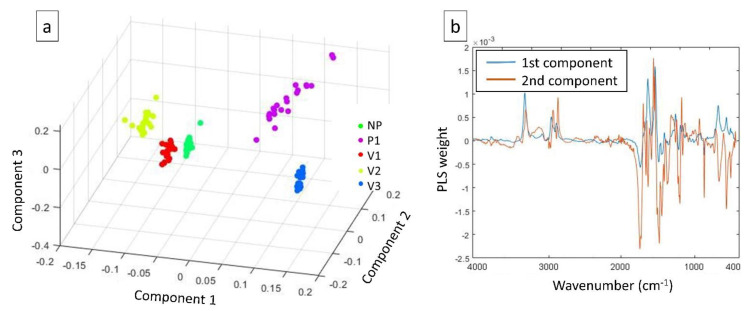
(**a**) Partial least squares-discriminant analysis (PLS-DA) 3D score plot; (**b**) PLS coefficients plot.

Similarly, the separation among the train dataset samples belonging to five types of polyamides was clear in the score plot of PLS-DA in Figure 5a, which also showed a reasonably narrow variability within each sample cluster (except for P1). PLS creates components (latent variables, LVs) by also taking into consideration the five different polyamide groups, resulting in better clustering. This outcome suggests that PLS can provide a more suitable discrimination model among the five classes than PCA. Assumptions for the built PLS-DA model are presented in the Supplementary Materials. The regression coefficients plot of the first two LVs (Figure 5b) showed that most of the spectral range is involved in the clustering of the polyamide samples, although the region of amide I and II seemed to be particularly responsible for the materials differentiation. However, from previous studies, it was found that using the whole spectral range is more advantageous since it brings more information about the functional group’s presence and interactions within and among the polymer chains [45].

**Figure 6:**
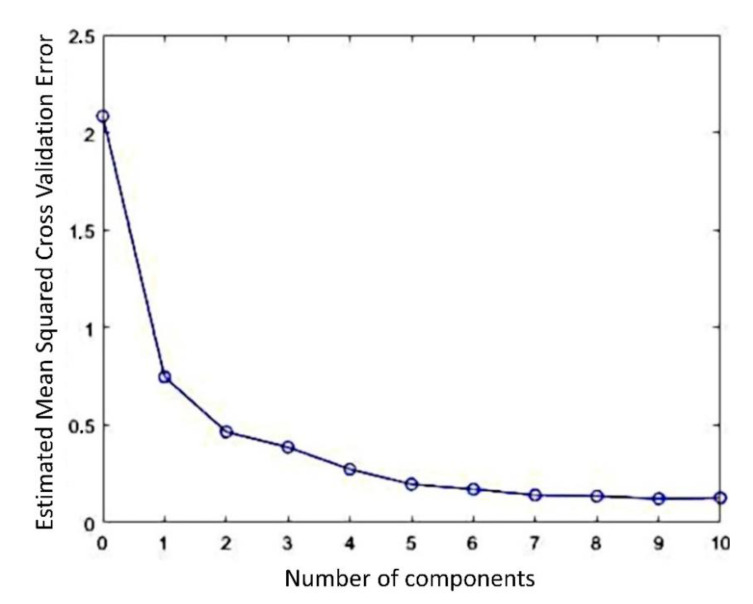
MSE of cross-validation (MSECV) indicating the number of components that should be used for the predictive PLS model.

As mentioned (Materials and Methods Section), the best prediction model to minimize the expected error without overfitting effect was evaluated as the lowest MSECV value in relation to the number of components, as observable in Figure 6. From these results, the number of seven components was chosen for the predictive PLS model.

**Figure 7:**
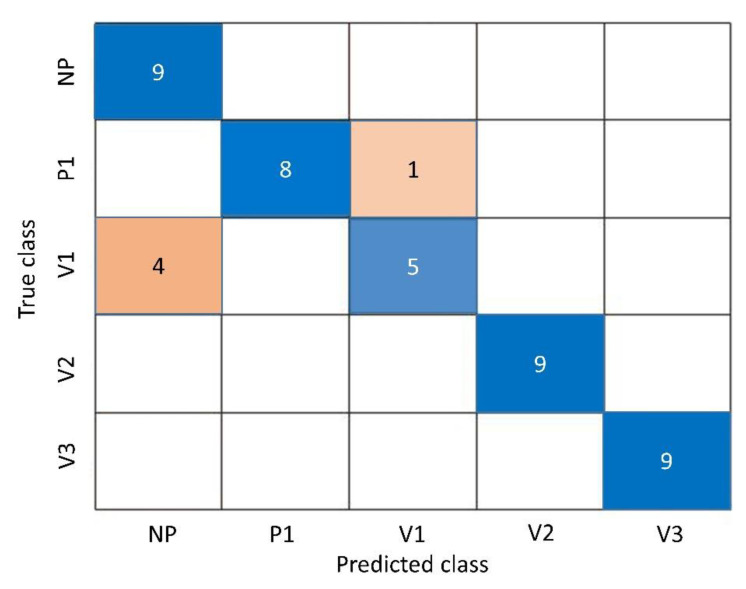
Confusion matrix of the predictive PLS model. Blue: samples where the actual class is positive, and the model correctly predicted it as positive. Red: samples where the actual class is negative, but the model incorrectly predicted it as positive, and vice versa. Darker shades of each color indicate a higher number of samples.

The 7-component PLS model was evaluated by the confusion matrix of the test dataset, as shown in Figure 7. The model presented a great classification of “unknown” samples with a total accuracy of 88.89%. It only mistakenly classified 5 samples of the polyamides P1 (one) and V1 (four) out of the total 45 samples of the test dataset, but the percentage of correct classification was high, ranging from 88.89% (P1) to 55.56% (V1). For the sake of clarity, only P1 and V1 samples seemed to be the most critical samples to be distinguished, presumably since they fall in a small range of viscosities and have very similar features. On the other hand, it provided 100% correct classifications for NP, V2, and V3. Prediction research does not provide thresholds for accuracy and overfit [57]. Indeed, increasing the number of components in the model can provide higher accuracy, but overfitting will be present [57]. Overall, the PLS model gave worthwhile sensitivities and specificities when assisted the ATR-FTIR technique in segregating different polyamides 6.9, as observable in Table 3.

In order to interpret the spectral differences detected by chemometrics, we deepened the meaning of some ATR-FTIR signals. Starting from the amide I (AI) and amide II (AII) signals recognized as particularly significant (see Figure 5b), the height and area ratios between the AI and AII peaks for each sample were calculated. If the ratios between the areas were coincident in all the cases, the ratios between AI and AII peak heights slightly variated, following the order V3 (1.6) > P1 (1.5) > NP = V2 (1.4) > V1 (1.2), where the values at the extremes correspond to the samples V3 and V1 that have the largest VR difference. This is explainable by the fact that amide I and II bands are sensitive to structural changes and chemical environment modifications owing to their propensity to form the polyamide H-bond network [58,59,60]. Moreover, the higher the viscosity, the more links are present within the chain than terminal groups, which mainly determine the extent of amino-related signals.

**Figure 8:**
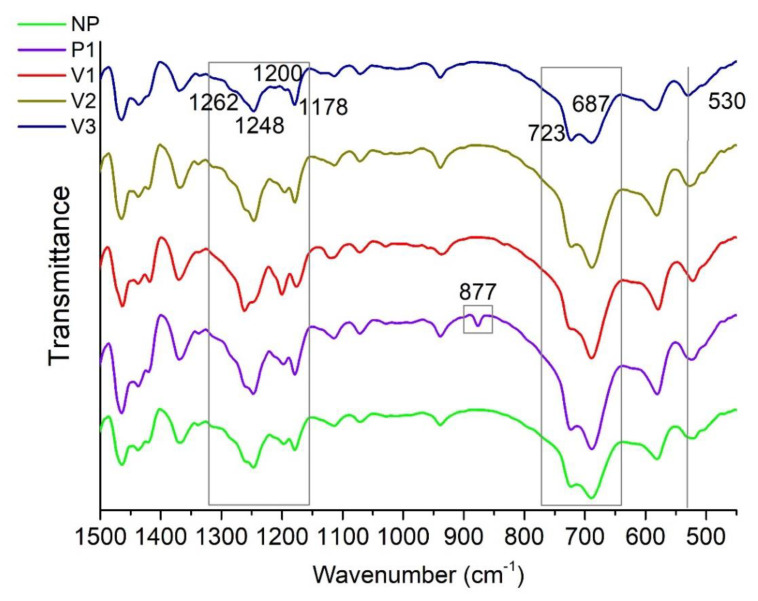
Zoom of the FTIR spectra from 450 to 1500 cm^−1^.

Moreover, Figure 8 shows other areas that bring forth some considerations. The peaks at 1262 and 1248 cm^−1^ and their relative ratios are similar for the samples NP, P1, and V2, while in V3, the signal at 1262 cm^−1^ becomes a shoulder, and in V1, the peaks’ intensity ratio appears inverted. The same two samples (V3 and V1) also demonstrated that they are different in relation to the peak couples 1200–1178 cm^−1^ and 723–687 cm^−1^. These discrepancies can be attributed to the crystallization pathway that the two materials with the highest RV differences underwent during their synthesis, presumably causing small modifications in the crystalline structure (i.e., α and γ forms relationships) [16,46,55,61], but not directly detected by DSC. Regarding the change of shape and the slight shift along the sample series for the peak centered at 530 cm^−1^ for V3 (skeletal bending), it is possible to hypothesize that these phenomena are related to the diverse needed energies for backbone vibrations, implying different order and chain conformations [47]. If, on the one hand, the most important discrimination causes are attributed to the viscosity differences, on the other hand, the purification procedure has no driven significant spectral modifications, except for the appearance of the peak at 877 cm^−1^. Pagacz et al. [62], who studied bio-polyamides based on renewable raw materials, assumed that this signal could be assigned to an amorphous contribution, but it could also derive from partial oxidation (C–O–O– vibrations). Analogously, Porubská et al. [63] detected the same absorption band in the spectrum of an irradiated polyamide 6, attributing it to alkyl peroxides. Since the peak appeared in the case of the only sample whose originating monomer salt had been specifically manipulated before the polymerization, we can suppose that the treatment has somehow contributed to partial oxidation phenomena.

## 4. Conclusions and Perspectives

ATR-FTIR is a fast, non-destructive, and inexpensive spectroscopic technique for the recognition of chemical compounds. The synergy between the acquisition of IR experimental data and chemometrics can lead to the development of an objective method for the identification and distinction of materials of a very similar nature, representing potential support to the quality control activities within the production sectors, for instance, as a first screening procedure. In this paper, PA69 samples containing a different amount of impurities or characterized by diverse viscosity values were examined. The hybrid approach of ATR-FTIR/chemometrics was more convenient than traditional analyses such as infrared spectroscopy (alone), differential scanning calorimetry, or mechanical tests, which, in the presence of the same chemical composition, did not give specific information to identify sample differences quickly. More in-depth, it was hypothesized that, although the whole spectral range brought more comprehensive information with respect to single spectral regions, the differences that could be seen seemed more related to the modified chemical environment caused by viscosity variation, both in terms of methylene vibration modes and hydrogen bonds. Although this work has been designed for the preliminary quality control of polymers, the methodology can be extended to other kinds of compounds.

## Figures and Tables

**Table 1:** List of samples and their main features. NP stands for non-purified, whereas P1 stands for purified, and V1, V2, and V3 are the non-purified samples characterized by increasing relative viscosity.

Sample Label	Colour, b *	Relative Viscosity
NP	11	2.57
P1	2.5	2.62
V1	14	2.34
V2	13	3.23
V3	6	3.50

**Table 2:** Main physical and mechanical properties of NP and V2.

Sample	NP	V2
Melting Point (°C)	213	214
Melt Volume Rate (cm^3^/10 min)	35.3	9.7
Notched Izod (kJ/m)	0.032	0.044
Unnotched Izod (kJ/m)	2.2	1.9
Notched Charpy (kJ/m^2^)	4.9	5.8
Unnotched Charpy (kJ/m^2^)	224	180
Flexural Modulus (GPa)	1.5	1.7
Tensile Strength (MPa)	57.0	56.5
Strain at yield (%)	4.6	4.6
Elongation at break (%)	141	140

**Table 3:** Sensitivity and specificity of different polyamide groups, calculated from the confusion matrix.

Polyamide Groups	Sensitivity	Specificity
NP	1	0.89
P1	0.89	1
V1	0.56	0.97
V2	1	1
V3	1	1

## Data Availability

All data supporting the findings of this study are available within the article.

## Supplementary Materials

The following supporting information can be downloaded at: https://www.mdpi.com/article/10.3390/polym15153166/s1, Figure S1: Explained variance vs. the number of PCs on principal component analysis; Figure S2: Heat map of correlation matrix among the measured variables; Figure S3: Heat map of covariance matrix among the measured variables; Figure S4: Fitted vs. Observed responses plot of 7-components PLS-DA model; Figure S5: Residuals vs. fitted values plot of the PLS-DA model; Figure S6: Scale-location plot of the PLS-DA model; Figure S7: Histogram of residuals of the PLS-DA model; Figure S8: Q-Q plot (Quantile–Quantile plot) of the PLS-DA model; Table S1: Test for normality Using Royston’s test and Mardia’s test.
