# Supplementary material for: Configuration of a Simple Method for Different Polyamides 6.9 Recognition by ATR-FTIR Analysis Coupled with Chemometrics
Source: Polymers (Basel). 2023 Jul 26;15(15):3166. doi: 10.3390/polym15153166 (PMC10420875; doi:10.3390/polym15153166)
Supplement: Supplementary file 1 [file polymers-15-03166-s001.zip › polymers-2441260-supplementary.pdf]

Principal Component Analysis (PCA)

PCA was performed based on the measured infrared spectra (4000-450 cm^{-1}), obtaining 1776 variables. The seven components that constructed the model explained 97% of the total variance among the polyamide types (Fig. S1). The correlation among variables was studied and results were visualized as Heat-map in Fig. S2. In addition, the Heat-map of variance-covariance matrix is depicted in Fig. S3.

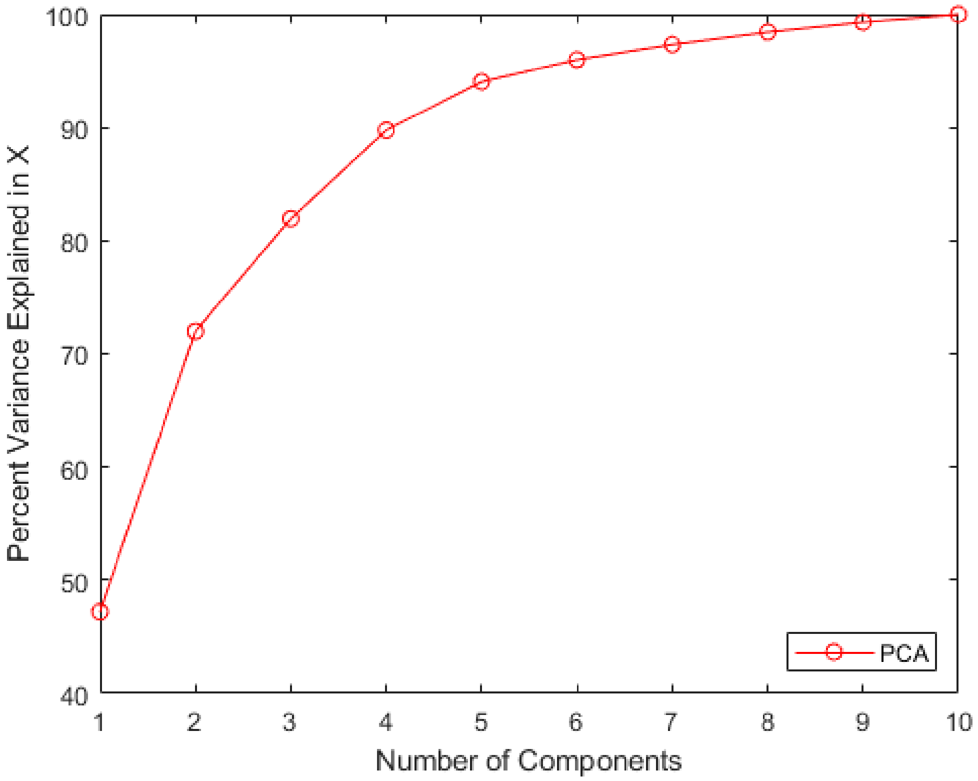

Figure S1. Explained variance vs. the number of PCs on principal component analysis.

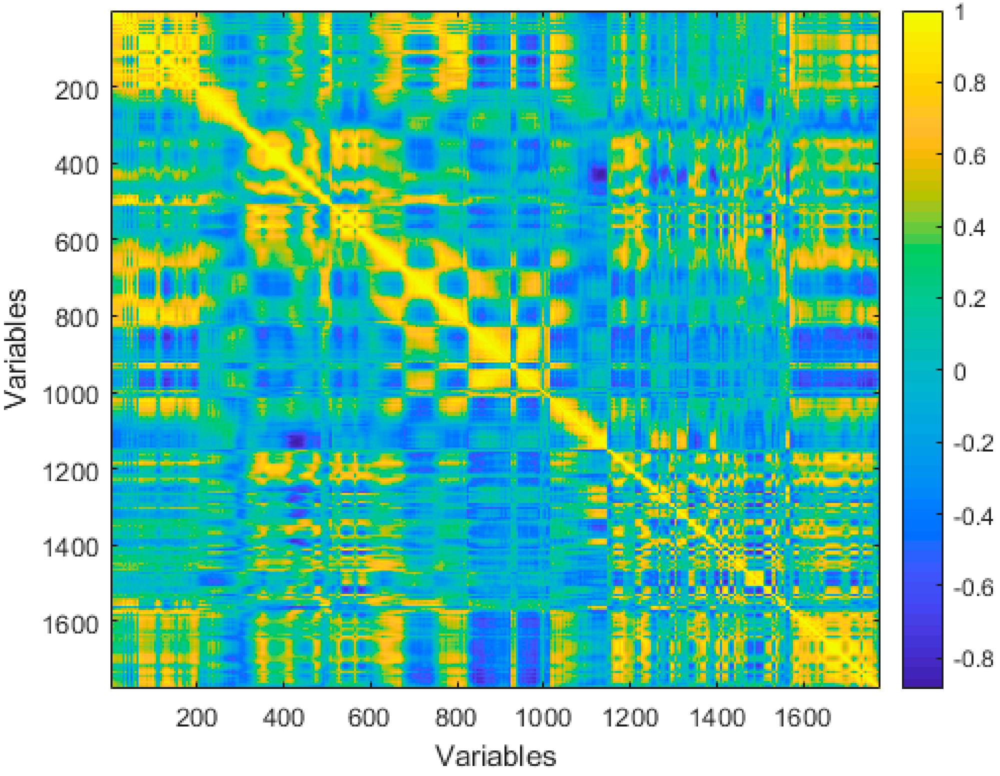

Figure S2. Heat map of correlation matrix among the measured variables.

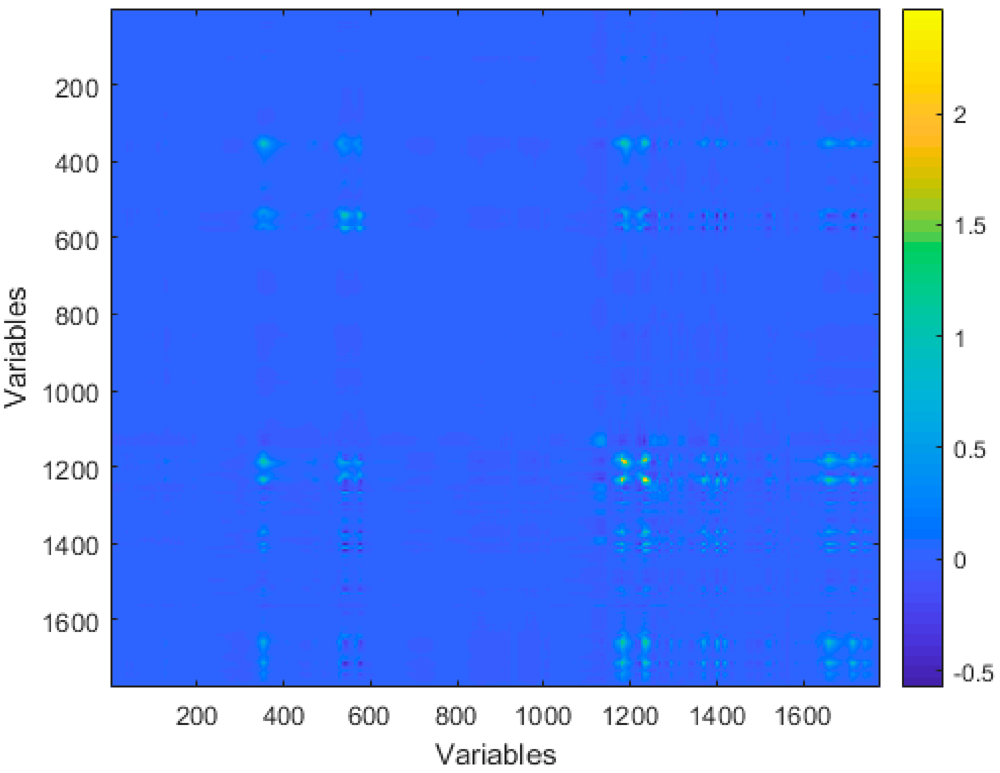

Figure S3. Heat map of covariance matrix among the measured variables.

PLS-DA

Taking into consideration the fact that PLS-DA model is a supervised method and it demonstrates better fitting ability, the rest of the diagnostics were run only for the PLS model, which was the chosen one for the discrimination of the study.

Since the proposed PLS-DA model must be robust enough, we investigated for statistical evidence to prove model robustness. Consequently, assumptions of linearity, homoscedasticity, normality and independence were studied.

To begin the analysis PLS-DA model was fitted and the observed vs. fitted values plot was computed (Fig. S4). According to the R^2 of the model (0.9516), PLS shows excellent goodness of fit, without foggy or dispersed points.

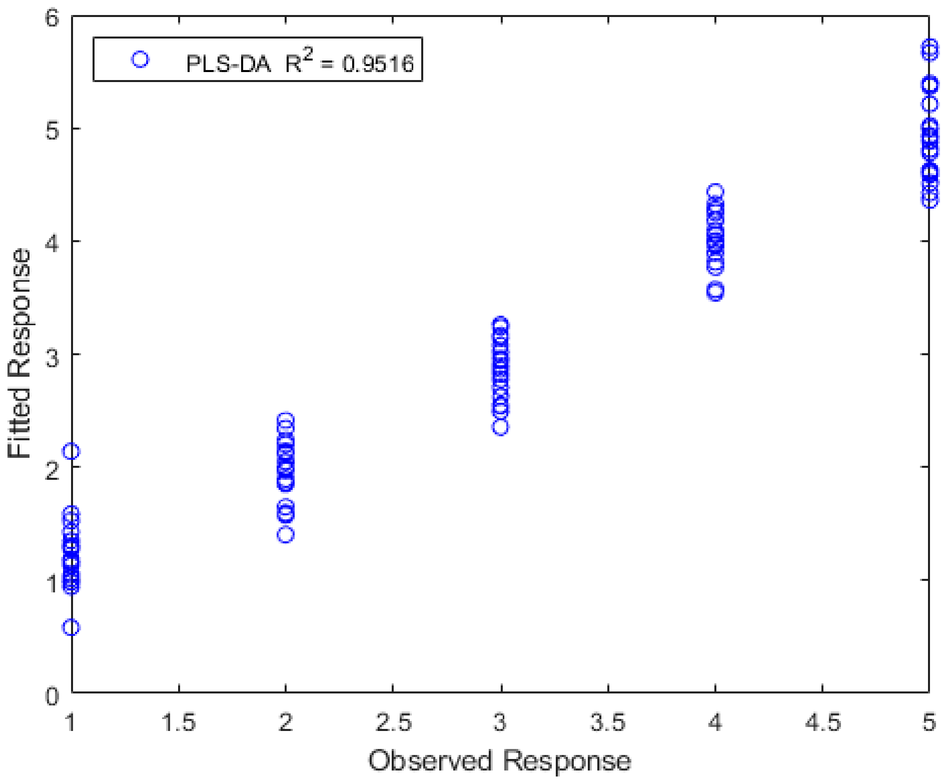

Figure S4. Fitted vs. Observed responses plot of 7-components PLS-DA model.

The linearity of the model was assumed via residuals vs. fitting responses plot. Since the analysis contained categorical values, the plot (Fig. S5) shows a pattern of diagonal lines. The residuals are scattered randomly and roughly form a "horizontal band" (red line) around the 0 line. Inspecting Figure S5, it is obvious that there is no fitted pattern which suggests that the data present a linear behavior.

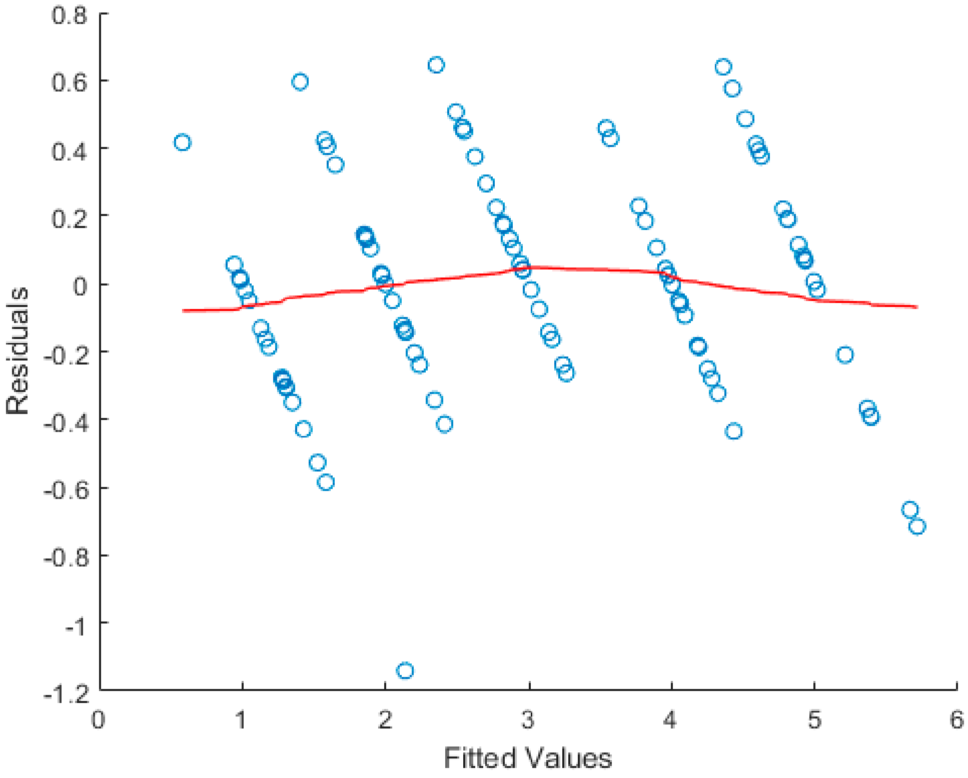

Figure S5. Residuals vs. fitted values plot of the PLS-DA model.

Being conceded for heteroscedasticity scenarios, the scale-location plot was studied (Fig. S6). This plot indicated that the variability (variances) of the residual points is constant with the value of the fitted outcome variable, suggesting constant variances in the residual errors (or homoscedasticity).

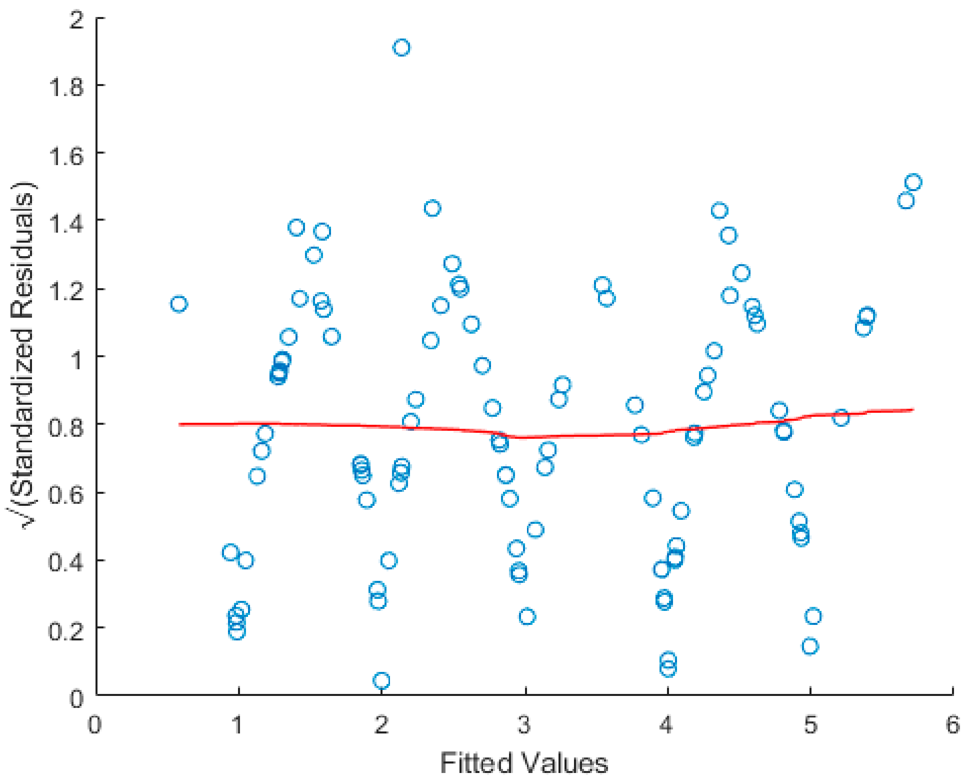

Figure S6. Scale-location plot of the PLS-DA model.

In order to assess the normality assumption, a histogram of residuals was examined (Fig. S7). A normal distribution would typically show a symmetrical bell-shaped curve, without any type of skew, indicating that the residuals follow a normal distribution, exactly as shown in Fig. S7. In addition, a Q-Q plot was generated in order to double-check the normality of the model (Fig. S8). The Q-Q plot indicates a straight-line pattern of the data, suggesting that the data closely resembled a normal distribution. Based on this, the research team concluded that there were no apparent violations from the normality assumption.

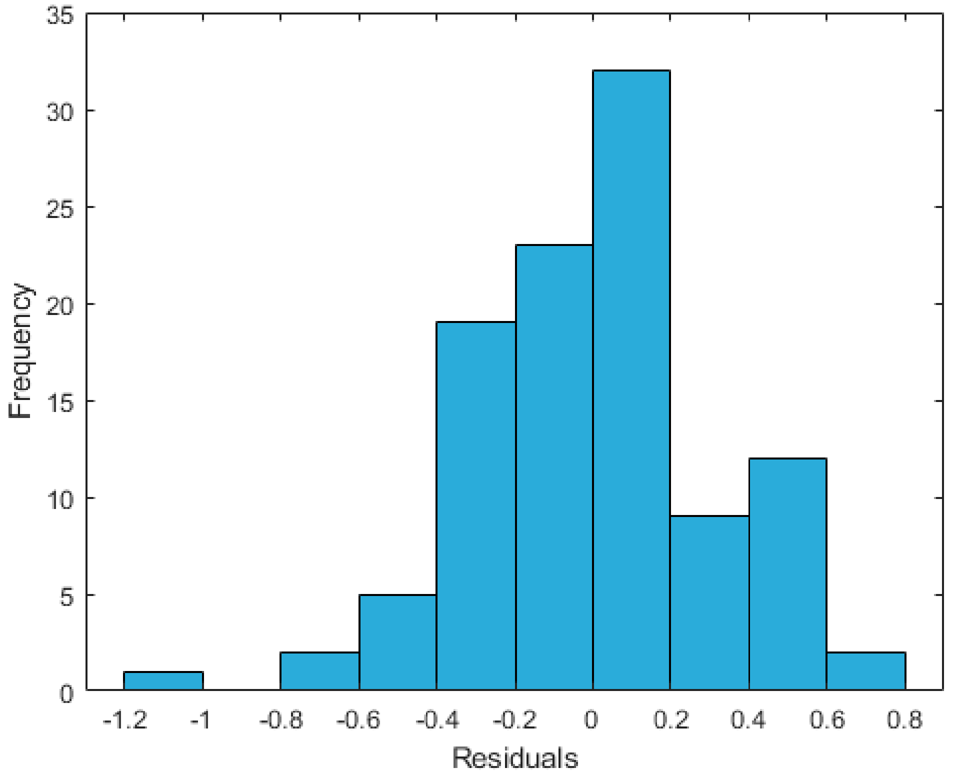

Figure S7. Histogram of residuals of the PLS-DA model.

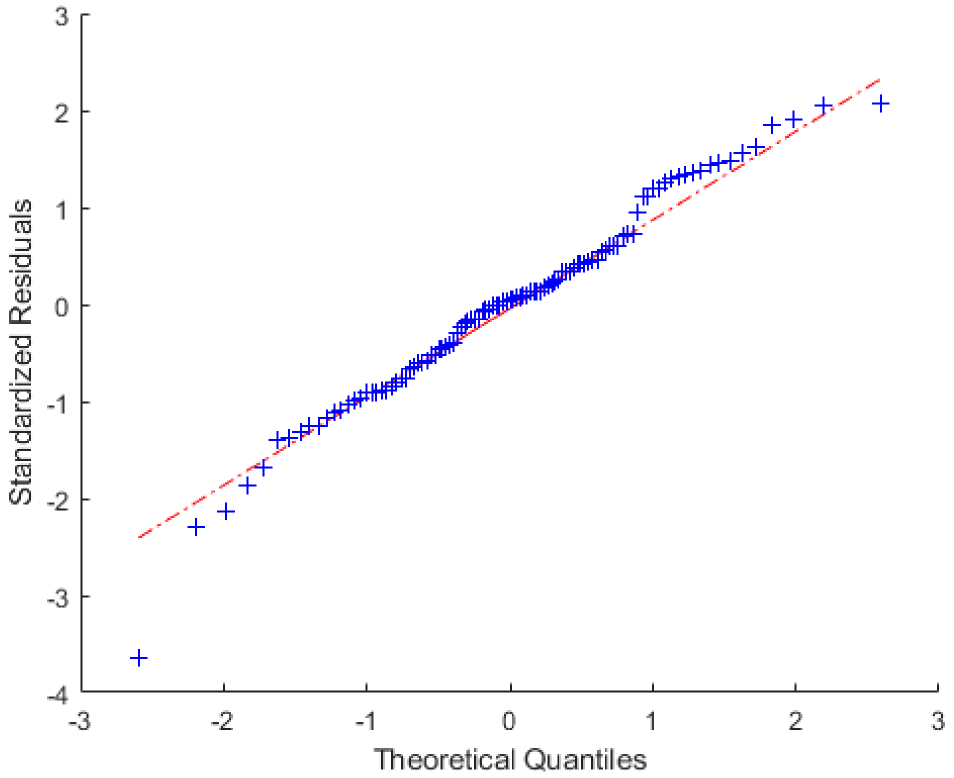

Figure S8. Q-Q plot (Quantile–Quantile plot) of the PLS-DA model.

Components are often seen as separate and distinct identities, and in most cases are interpreted based on this assumption. However, it's important to note that uncorrelated PCs or LVs are truly independent only when the assumption of multivariate normality holds true [1]. If the normality assumption is violated, uncorrelated components may not necessarily be independent. Several tests have been proposed for testing multivariate normality, in our study two of the most commonly used tests namely; Royston's test and Mardia's test [2] were employed (see Table S1).

Table S1. Test for normality Using Royston's test and Mardia's test

Test	Royston’s	Mardia's
Test statistic	0.068898	0.0017895
p-value	0.79295	0.96626

The results obtained from both tests confirm multivariate normality (and as a result components are truly independent) since the p-values for both are far greater than the significance level of 0.05

References

1. Zhou, M.; Shao, Y. A Powerful Test for Multivariate Normality. *J. Appl. Stat.* **2014**, *41*, 351–363, doi:10.1080/02664763.2013.839637.
2. Boakye Oppong, F.; Yao Agbedra, S. Assessing Univariate and Multivariate Normality, A Guide For Non-Statisticians. *Math. Theory Model.* **2016**, *6*, 26–33.
